# Experimental Test for Facilitation of Seedling Recruitment by the Dominant Bunchgrass in a Fire-Maintained Savanna

**DOI:** 10.1371/journal.pone.0039108

**Published:** 2012-07-06

**Authors:** Gwenllian D. Iacona, L. Katherine Kirkman, Emilio M. Bruna

**Affiliations:** 1 J. W. Jones Ecological Research Center, Newton, Georgia, United States of America; 2 Department of Wildlife Ecology and Conservation, University of Florida, Gainesville, Florida, United States of America; 3 Center for Latin American Studies, University of Florida, Gainesville, Florida, United States of America; USDA-ARS, United States of America

## Abstract

Facilitative interactions between neighboring plants can influence community composition, especially in locations where environmental stress is a factor limiting competitive effects. The longleaf pine savanna of the southeastern United States is a threatened and diverse system where seedling recruitment success and understory species richness levels are regulated by the availability of moist microsites. We hypothesized that the dominant bunch grass species (*Aristida stricta* Michx.) would facilitate moist seedling microsites through shading, but that the effect would depend on stress gradients. Here, we examined the environmental properties modified by the presence of wiregrass and tested the importance of increased shade as a potential facilitative mechanism promoting seedling recruitment across spatial and temporal stress gradients. We showed that environmental gradients, season, and experimental water manipulation influence seedling success. Environmental properties were modified by wiregrass proximity in a manner that could facilitate seedling success, but we showed that shade alone does not provide a facilitative benefit to seedlings in this system.

## Introduction

Facilitative interactions occur when a plant enhances the success of another [1]. Because these relationships can influence patterns of community composition, identifying the extent of facilitation and its underlying mechanisms is important for understanding the drivers of plant diversity [2]. Facilitation in plant communities appears to be relatively common, particularly in stressful systems where the benefits provided by a neighboring plant outweigh competitive interactions [2], [3], [4], [5]. However, the nature of the interaction may vary across a continuum determined by environmental stress gradients, with competitive effects dominating in optimal conditions and facilitation prevailing in harsher ones [2], [3], [6]. These stress gradients may be temporal (e.g. inter-seasonal differences [7]), or spatial (e.g., gradients in soil moisture [4], or salinity [8]).

Facilitative interactions can influence plant community composition by various mechanisms including those that enhance seedling success. The nutrient uptake, transpiration, and physical presence of a “nurse plant” changes the local microenvironment and may impact nearby seedlings [1]. These effects can enhance nutrient supply [9], [10], or increase water availability for a seedling, and can be particularly important early in establishment or during drought [11], [12]. In addition, nurse plants can facilitate recruitment by modulating temperature highs and lows beneath their canopy [13].

Despite their putative importance, the role of facilitative interactions in the maintenance of species diversity is still unclear [2]. In species-rich systems, facilitative habitat amelioration or increased resource availability could expand the fundamental niche of a species and thus explain patterns of coexistence that cannot be fully explained by competitive exclusion [6], [14]. One species-rich system that could be used to test for facilitative drivers of species diversity is the longleaf pine (*Pinus palustris* Mill.) savanna of the southeastern United States [15]. Previously we have shown that water availability regulates diversity of seedling species in this system, which suggests that heterogeneity in the availability of moist microsites influences community composition [16]. It is possible that the presence of such microsites is a consequence of plant-plant interactions. There is currently no evidence that positive plant-plant interactions play a role in promoting small scale species richness in this system. However, the observed positive correlation between understory species diversity and annual net primary productivity may indicate a beneficial relationship between plant biomass and seedling establishment [17]. In addition, the longleaf pine savanna is characterized by a variety of stresses including low water and nutrient availability. These two factors likely interact with frequent fires and high summer temperatures to limit competitive influences on seedling recruitment, suggesting that the conditions are appropriate for facilitation to occur [18], [19].

Wiregrass (*Aristida stricta* Michx.) is the dominant understory species in the southeastern extent of the longleaf pine savanna [20], and it forms a near continuous groundcover in the regions where species richness is highest [21]. There are several mechanisms by which the presence of wiregrass could affect seedling success. The primary facilitative benefit may be enhanced seedling water retention from reduced evapotranspiration in the shade [11], as has been demonstrated in some arid ecosystems [22], [23]. Alternatively, elevated soil carbon pools beneath clumps of wiregrass [24] could enhance soil water holding capacity [25]. Finally, the non-rhizomatous structure of wiregrass with scattered clumps and numerous arching wiry leaves may protect seedlings from high temperatures and other environmental extremes [26].

Here, we assessed variation in seedling microsites, and tested shade as a mechanism to enhance seedling recruitment across a moisture stress gradient. We characterized the abiotic environment of potential seedling recruitment microsites located under and between wiregrass clumps. We also used seed and resource manipulations to determine how shade, and the interaction of shade and water availability, influenced seedling recruitment. The experiment incorporated a naturally occurring gradient in soil moisture and a seasonal gradient over two years. We predicted that the facilitative effects of shading should be more important in xeric communities and after the summer months where temperature and moisture stress is more apparent. Also, we expected that adding water would reduce the effect of facilitation, and the effect of water addition would be more apparent in xeric locations (observable as an interaction).

## Methods

### Study Site

Ichauway (31° 13′ N, 84° 29′ W) is an 11,300 ha property of the Joseph W. Jones Ecological Research Center that includes 7,500 ha of longleaf-pine savanna. A wiregrass-dominated groundcover that is characterized by notably high levels of fine scale species richness [27] has been managed under a 2–5 year prescribed fire return interval for at least the past 90 years. This site is located in the Lower Coastal Plain and Flatwoods (LCPF) section of southwest Georgia, USA [28]. Average yearly temperatures range from 5–17°C in the winter to 21–34°C in the summer with about 131 cm of precipitation evenly distributed throughout the year [29]. Across its range, the longleaf pine-wiregrass savanna occupies a wide moisture gradient that extends from extremely mesic locations with saturated soils and a perched water table to extremely xeric locations along deep sand ridges. At Ichauway, the wiregrass dominated understory extends from mesic areas with seasonally saturated Aquic Arenic Paleudult soils to xeric areas with Typic Quartzipsamments [29].

### Microhabitat Properties

To compare environmental conditions in potential seedling recruitment microsites that differed in their proximity to wiregrass, we measured soil and air temperature, soil moisture, relative humidity, and light availability beneath and adjacent to naturally occurring wiregrass clumps from April to August, 2006. In each control plot (as described below), we randomly selected a site under a wiregrass clump (hereafter, “below”) and identified the nearest bare soil patch with no wiregrass canopy (hereafter, “between”). At each of these microsites (n = 16), we estimated volumetric soil moisture at 10 cm depth using time-domain reflectometry (TDR) [30]. For this procedure, we installed a pair of 11 cm stainless steel rods vertically at each microsite to quantify soil moisture three days per month using a Techtronix cable tester. We measured photosynthetically active radiation (PAR µmol.m^−2^.s^−1^) at ground level at each microsite with a quantum line sensor ceptometer (Accupar LP80, Decagon Devices, Pullman WA). Readings above the wiregrass canopy were obtained simultaneously at 50 cm above the ground using an external PAR spot sensor. For each observation, we recorded three consecutive PAR readings and all observations were made between the hours of 10∶00 AM and 2∶00 PM on sunny days. We recorded microsite air temperature and relative humidity at 5 cm above ground level with multimode data loggers (HOBO pro H8, Onset Computer Corporations, Bourne MA). We also measured soil temperature at a depth of 5 cm using one four channel data logger (HOBO H8, Onset Computer Corporations, Bourne MA) per plot with two temperature probes on leads so that measurements could be obtained at both microsite locations simultaneously. All data loggers took readings at one hour intervals for 72 consecutive hours each month.

### Resource Treatments

Our experiments took advantage of an ongoing and long-term experimental resource manipulation established at Ichauway in 2002. We manipulated water and shade at both the mesic and xeric ends of the natural soil moisture gradient (mean percent baseline volumetric soil moisture 8±0.68 in xeric, and 15±1.04 in mesic). We had eight randomly assigned water addition plots (50 m×50 m) and eight randomly assigned control plots split between the soil moisture extremes in a fully factorial 2×2 experimental design. Irrigation with approximately 825 mm of water per year (∼65% increase over yearly precipitation) was ongoing since 2002 and maintained the water treatment plots at close to 40% field capacity. This level of water addition was determined by engineering limitations, but it was a substantial increase for the system and thus was likely to remedy the water limitations that regulate seedling recruitment (R. Mitchell, *personal communication*) [17]. Irrigation was applied for 24 hours once every eight days unless sufficient rain fell to reach the target field capacity. To minimize cation accumulation in the soil, water was treated with reverse osmosis.

To simulate the effects of shading by wiregrass, and to examine the potential importance of interactions of shade and water availability on seedling recruitment rates, we constructed shade cloth structures over experimentally sown seedling communities replicated within the plots (n = 2 per plot). We used a split-split plot design that manipulated shade levels in control and watered plots at the wet and dry extremes of the naturally occurring moisture gradient for two growing seasons.

In 2003, we installed 4 poly vinyl chloride (PVC) rings (30 cm diameter×10 cm deep) in each of the 16 plots. These rings served as seedling recruitment manipulation sites and allowed us to ensure that observed seedlings were recruits from seed rather than vegetative resprouts. To install the PVC, we first hammered a 30 cm steel ring into the soil to sever the plant roots then extracted this ring with as little soil disturbance as possible. We inserted the PVC rings into the pre-cut trench so that the top edge of the ring was flush with the ground. In 2005, we removed the existing vegetation in each ring with two treatments of glyphosate, applied in summer and fall. All sites were burned in February 2004 and 2006.

In March 2006, we installed shade structures over two randomly selected rings in each plot and left two rings unshaded (n = 64 rings). For the first year of the experiment, multiple layers of shade cloth and screen were fitted to wooden frames (55 cm×55 cm×30 cm tall) and reduced light at the soil surface by 90%. This level of shade was chosen to approximate the light reduction provided by wiregrass cover (mean ±1SD  = 78±1%, max 99%; R. Mitchell, *unpubl. data*). Due to low overall recruitment in 2006, we adjusted the shading to 80% for the second study year. Between March and May 2006, and again in March 2007, we sowed 50 seeds per ring of each of the following species: *Sorghastrum secundum*, *Rudbekia hirta* and *Desmodium ciliare*. We chose these species because they are common grass, forb, and legume species in the longleaf pine understory, and are known to germinate under field conditions. All seeds were obtained from the native plant research garden at Ichauway. We determined the percent viability of each species using petri dish germination or tetrazolium tests in 2007 (*R. hirta*: 75% viable, *S. secundum*: 40% viable, *D. ciliare*: 90% viable). To reduce loss of seeds due to wind, we fitted an 8 cm tall ring of aluminum window screen around each unshaded PVC ring. We counted all seedlings of each species in May and November 2006 and 2007.

### Statistical Analyses: Microhabitat Properties

We tested for differences in environmental parameters at seedling microsites that differed with in proximity to wiregrass clumps using a mixed model procedure (PROC MIXED, SAS Institute Inc. Version 9.1). The mean value per plot per month was analyzed for each month, with plot treated as a random effect, and proximity to wiregrass (below or between) considered a fixed effect. To observe fine scale variation, we analyzed mesic and xeric locations by month to minimize the variability between locations and over time. Statistical significance was established at α = 0.05.

### Statistical Analyses: Resource Treatments

We used a mixed model analysis to test for mean differences in recruitment rate attributable to shade, water availability, collection period, moisture gradient location and for interactions between shade, water availability and moisture gradient location. Due to low numbers of recruits, we grouped seedlings of all three species together for analysis. Preliminary analysis did not show any variability in recruitment response across species (May 2006, treatment×species interaction effect test; F = 0.07, Pr>F = 0.93). We compared the mean number of seedlings in shaded and unshaded treatments (two rings of each per plot) across watered and control plots at mesic and xeric gradient locations over four collection periods using a split-split plot design (n = 32, PROC MIXED, SAS Institute Inc. Version 9.1). Shade treatment (split-split plot factor), water manipulation (whole-plot factor), gradient location (whole-plot factor) and collection period (split-plot factor) were modeled as fixed effects, and plot was modeled as a random effect. We included collection period as a split-plot factor rather than using a repeated measures framework since the number of seedlings was assumed to increase, but we were interested in the interaction between time and resource availability. We used planned contrasts of combinations of collection period to examine seedling response to season and year. The model was fit using a Gaussian distribution because the use of mean response values allowed for the assumption of a normal distribution. In addition, examination of the model residuals indicated adequate model fit. Statistical significance was assessed at α = 0.05.

## Results

### Microhabitat Properties

**Figure 1:**
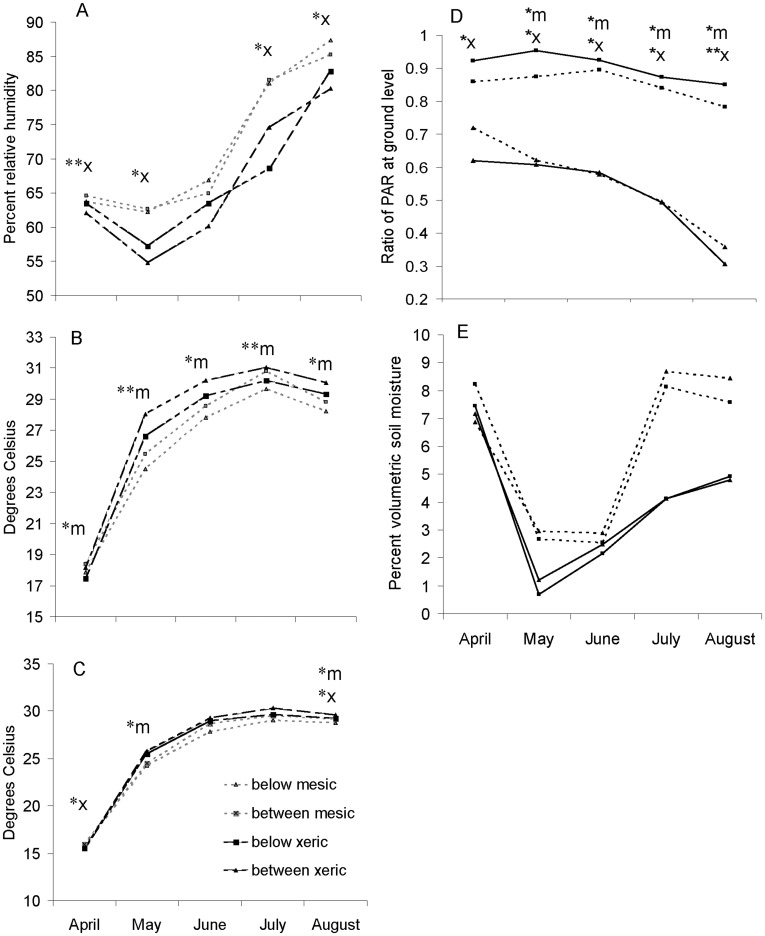
Microsite measurements. Values represent environmental measurements at potential seedling microsites below and between clumps of wiregrass; (A) relative humidity, (B) soil temperature, (C) air temperature, (D) photosynthetically active radiation, (E) volumetric soil moisture. Differences between microsite locations are indicated by (m) or (x) for mesic or xeric location respectively (* = p≤0.05, ** = p≤0.08). Refer to Table 1 for mean factor values.

The magnitude of difference in environmental factors “below” versus “between” wiregrass clumps ranged from 30% variation in PAR to almost no variation in volumetric soil moisture (Table 1). Shading (reduction in PAR) was consistently greater under wiregrass clumps (Figure 1d). Meanwhile, differences between microsites with respect to relative humidity, soil temperature, and air temperature values were usually small and dependent on gradient location and month. Volumetric soil moisture did not vary with microsite proximity, season, or gradient location (Figure 1e).

**Table 1:** Mean environmental factor values for microsites below wiregrass clumps and microsites between wiregrass clumps.

Location	Measure	Least square mean below ± SE			Least square mean between ± SE		
Mesic	Relative Humidity (%)	72.84	±	2.82	72.63	±	2.68
	Soil Temperature (°C)	25.24	±	1.01	26.04	±	1.15
	Air Temperature (°C)	24.86	±	1.30	25.19	±	1.33
	Incident PAR (proportion)	0.55	±	0.04	0.85	±	0.02
	Volumetric Soil Moisture (%)	6.10	±	0.76	6.01	±	0.76
Xeric	Relative Humidity (%)	69.52	±	2.82	67.06	±	2.82
	Soil Temperature (°C)	26.26	±	1.25	27.20	±	1.31
	Air Temperature (°C)	25.47	±	1.41	25.77	±	1.44
	Incident PAR (proportion)	0.52	±	0.04	0.91	±	0.04
	Volumetric Soil Moisture (%)	4.19	±	0.55	4.19	±	0.62

The values of all factors varied across months, but there was no clear temporal pattern in difference for microsites between versus below wiregrass clumps. Meanwhile, differences between microsite types for some factors were related to gradient location. In the xeric location, percent relative humidity was significantly or marginally (p≤0.08) greater below wiregrass clumps for three of the five months. No difference occurred with proximity to wiregrass in the mesic location (Figure 1a). In contrast, the average soil temperature was greater (or marginally greater) between than below wiregrass clumps for four of the five months at the mesic location, but never differed at the xeric location (Figure 1b). Finally, there was no clear pattern to variation in air temperature. It was slightly higher (0.4 C° difference) at microsites between wiregrass clumps only in May and August in the xeric location but differed in June and in August in the mesic location.

### Resource Treatments

**Figure 2:**
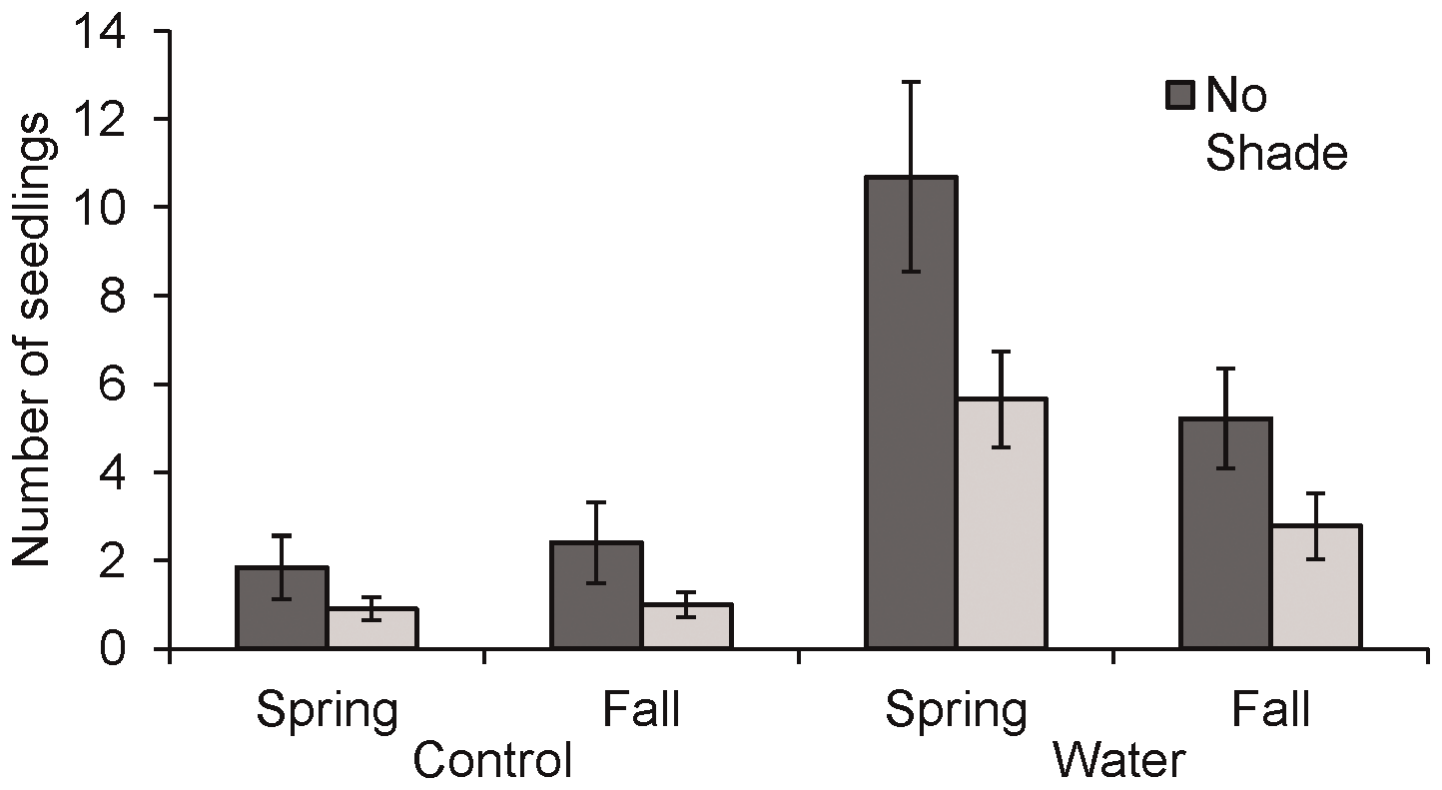
Seedling response to experimental shading and water addition. Values are mean number of seedling recruits in response to experimental manipulation of water availability and shade. Refer to Table 2 for analysis results and Table 3 for mean factor values.

We observed twice as many seedling recruits in unshaded treatment plots versus in shaded plots across all collection periods (Table 2, Table 3), and there were about four times more seedlings in watered plots than in unwatered plots (Figure 2, Table 2, Table 3). An interaction between water manipulation and shade treatment suggested that shading had a more negative effect on seedling recruitment in the control plots than in the watered plots (Table 3). Although the number of seedlings increased with time, an interaction with water availability led to greater recruitment in spring in irrigated sites (Table 2, Table3).

**Table 2:** Seedling recruitment analysis results.

Fixed Effects				
Effect	Num DF	Den DF	F Value	Pr > F
resource treatment	1	12	11.56	0.0053
collection event	3	36	4.77	0.0067
resource*collection	3	36	6.18	0.0017
location on gradient	1	12	0.40	0.54
resource*location	1	12	0.44	0.5181
collection*location	3	36	1.41	0.256
resource*collection*location	3	36	0.81	0.4943
shade treatment	1	48	14.23	0.0004
resource*shade	1	48	3.88	0.0546
collection*shade	3	48	1.05	0.3794
resource*collection*shade	3	48	0.66	0.5779
location*shade	1	48	1.07	0.3067
resource*location*shade	1	48	0.19	0.6673
collection*loc*treatment	3	48	0.04	0.9872
resource*collection*location*shade	3	48	0.17	0.9173
**Planned Contrasts**				
	**Num DF**	**Den DF**	**F Value**	**Pr > F**
spring v fall	1	36	8.73	0.0055
2006 v 2007	1	36	5.43	0.0255

Results of split-split plot analysis of seedling recruitment across two shade treatments (shade, no shade), two water treatments (water, control), two gradient locations (mesic, xeric) and four collection periods (spring and fall, 2006 and 2007).

**Table 3:** Seedling recruitment least square means.

		Least square mean ± SE		
resource treatment	Control	1.54	±	0.95
	Water	6.09	±	0.95
location on gradient	Mesic	3.39	±	0.95
	Xeric	4.23	±	0.95
shade treatment	Shade	2.59	±	0.74
	No Shade	5.04	±	0.74
collection event	May 2006	3.89	±	0.87
	Nov 2006	2.22	±	0.87
	May 2007	5.66	±	0.87
	Nov 2007	3.48	±	0.87
resource*collection	Control*May06	1.41	±	1.24
	Control*Nov06	1.81	±	1.24
	Control*May07	1.34	±	1.24
	Control*May06	1.59	±	1.24
	Water*May06	6.38	±	1.24
	Water*Nov06	2.63	±	1.24
	Water*May07	9.97	±	1.24
	Water*May06	5.38	±	1.24
resource*shade	Control*No Shade	2.13	±	1.05
	Control*Shade	0.95	±	1.05
	Water*No Shade	7.97	±	1.05
	Water*Shade	4.23	±	1.05

Least square means of seedling recruitment across two shade treatments (shade, no shade), two water treatments (water, control), two gradient locations (mesic, xeric) and four collection periods (spring and fall, 2006 and 2007).

## Discussion

Seedling establishment in the long-leaf pine savanna is thought to be influenced by the heterogeneous water limitations that characterize the system [16], [17]. Indeed, our results showed that seedling recruitment varied across stress gradients and that factors that relieve moisture stress enhanced seedling success. In concert with our observational findings, these results suggest that facilitation is feasible in the system. However, our study did not identify direct facilitative effects of shading as predicted.

Our expectation that water stress regulates recruitment success was supported based on our observation of greater seedling establishment at the mesic end of the natural moisture gradient and in watering treatments. In addition, the presence of an interaction between water and season suggested that a temporal effect existed where additional water was particularly important during establishment in the spring. Yet, despite the larger number of seedlings in watered plots in the spring, neither shade nor water was sufficient to sustain the new seedlings through the hot summer months of the study period.

The result that shading does not facilitate seedling recruitment and that recruitment was actually *lower* under shading treatments than under full sun is surprising given the intense sunlight and high soil surface temperatures in this ecosystem [31]. Moisture availability did appear to modulate the impact of shade to some extent given that there was less of a negative effect of shade on seedling recruitment when water was applied. Nevertheless, our prediction of an interactive temporal effect of shade and water was not supported.

Several conditions associated with our experiment may have influenced our results. First, the level of shade we provided was at the high end of the range observed in the field [31] and may have inhibited seedling growth. This may be an example of an upper bound on facilitative effects in response to extreme environmental stress as suggested in a recent theoretical model [32]. Second, it is unclear to what degree the shade structures may have inhibited water availability. Other studies in water limited systems have observed similar reductions in recruitment success when water availability was obstructed in concert with shade [33], [34]. Furthermore, the presence of the interaction term between shade and water treatments suggests that reduced moisture availability may be responsible for the negative effect of shade. In either case, it could be argued that our treatment combinations were outside the range of environmental amelioration where facilitation can counteract drought stress in seedlings [19] Additionally, although no spatial gradient effects were indicated by the mixed model results, inference in this component of the study is limited by the clumped configuration of plots within the gradient locations. It is also important to note that there were extreme drought conditions during the study period (NCDC average annual precipitation for GA is 127 cm, yet 2006–2007 average was 101 cm). In such dry, hot conditions, water limitation may have imposed similar constraints on seedling survival in mesic as in xeric locations, thus potentially masking an effect of the natural moisture gradient. Finally, there is a possibility that seed predators could be attracted to the shade structures and disproportionally reduced the number of seedlings in shaded locations [35]. Determining the influence of these confounding factors requires additional testing.

Our results suggest that heavy shading alone does not promote facilitation even though water is paramount for recruitment in this system. This evidence suggests that the previously observed relationship between wiregrass productivity and system species diversity may alternatively hinge on community wide effects of wiregrass presence rather than the impact of individual clumps. We show that microsites in close proximity to wiregrass clumps tend to be slightly cooler and more humid than those without a nearby wiregrass clump. When these small differences are extrapolated up to the community scale, one could argue that an area with a dense wiregrass understory could contain more microsites suitable for seedling recruitment than an area that had a sparse understory. In this situation, the overall effect of facilitation on community species composition could be significant (eg. [36]). These results also have implications for assessing the importance of positive plant-plant interactions in other systems. They suggest that there may be considerable variation in the grain of spatial and temporal effects and thus a much wider range of stress gradient conditions must be considered. This may explain why a predictable relationship has yet to be observed between stressors and facilitative effects over time and space [32], [33]. We conclude that identification of possible facilitative effects on recruitment in this system will require examination of a wider range of conditions than in our study, particularly with regards to a gradient of wiregrass cover and during periods of greater rainfall.

## References

[1] Callaway RM (1995). Positive interactions among plants.. Botanical Review.

[2] Brooker RW, Maestre FT, Callaway RM, Lortie CL, Cavieres LA (2008). Facilitation in plant communities: the past, the present, and the future.. Journal of Ecology.

[3] Freestone AL (2006). Facilitation drives local abundance and regional distribution of a rare plant in a harsh environment.. Ecology.

[4] Greenlee JT, Callaway RM (1996). Abiotic stress and the relative importance of interference and facilitation in montane bunchgrass communities in western montana.. The American Naturalist.

[5] Hacker SD, Bertness MD (1995). Morphological and physiological consequences of a positive plant interaction.. Ecology.

[6] Bruno JF, Stachowiz JJ, Bertness MD (2003). Inclusion of facilitation into ecological theory.. Trends in Ecology and Evolution.

[7] Kikvidze Z, Khetsuriani L, Kikodze D, Callaway RM (2006). Seasonal shifts in competition and facilitation in subalpine plant communities of the central Caucasus.. Journal of Vegetation Science.

[8] Hacker SD, Bertness MD (1999). Experimental evidence for factors maintaining plant species diversity in a New England salt marsh.. Ecology.

[9] Shumway SW (2000). Facilitative effects of a sand dune shrub on species growing beneath the shrub canopy.. Oecologia.

[10] Vinton MA, Burke IC (1995). Interactions between individual plant-species and soil nutrient status in shortgrass steppe.. Ecology.

[11] Holmgren M, Scheffer M, Huston MA (1997). The interplay of facilitation and competition in plant communities.. ecology.

[12] Zou CB, Barnes PW, Archer S, McMurtry CR (2005). Soil moisture redistribution as a mechanism of facilitation in Savanna tree-shrub clusters.. Oecologia.

[13] Shreve F (1931). Physical conditions in sun and shade.. Ecology.

[14] Hacker SD, Gaines SD (1997). Some implications of direct positive interactions for community species diversity.. ecology.

[15] Keddy PA, Smith L, Campbell DR, Clark M, Montz G (2006). Patterns of herbaceous plant diversity in southeastern Louisiana pine savannas.. Applied Vegetation Science.

[16] Iacona G, Kirkman LK, Bruna EM (2010). Effects of resource availability on seedling recruitment in a fire-maintained savanna.. Oecologia.

[17] Kirkman LK, Mitchell RJ, Helton RC, Drew MB (2001). Productivity and species richness across an environmental gradient in a fire-dependent ecosystem.. American Journal of Botany.

[18] Pecot SD, Mitchell RJ, Palik BJ, Moser EB, Hiers JK (2007). Competitive responses of seedlings and understory plants in longleaf pine woodlands: separating canopy influences above and below ground.. Canadian Journal of Forest Research-Revue Canadienne De Recherche Forestiere.

[19] Holmgren M, Scheffer M (2010). Strong facilitation in mild environments: the stress gradient hypothesis revisited.. Journal of Ecology.

[20] Peet RK, Allard DJ (1993). Longleaf pine vegetation of the southern atlantic and eastern gulf coast regions: a preliminary classification;; Tallahassee, FL, USA..

[21] Mitchell RJ, Kirkman LK, Pecot SD, Wilson CA, Palik BJ (1999). Patterns and controls of ecosystem function in longleaf pine - wiregrass savannas. I. Aboveground net primary productivity.. Canadian Journal of Forest Research-Revue Canadienne De Recherche Forestiere.

[22] Abd El Rahman AA, Batanouny KH (1965). Transpiration of desert plants under different environmental conditions.. The Journal of Ecology.

[23] MacMahon JA, Schimpf DJ (1981). Water as a factor in the biology of North American desert plants.. Water in Desert Ecosystems.

[24] West JB, Donovan LA (2004). Effects of individual bunchgrasses on potential C and N mineralization of longleaf pine savanna soils.. Journal of the Torrey Botanical Society.

[25] Olness A, Archer D (2005). Effect of organic carbon on available water in soil.. Soil Science.

[26] Fulbright TE, Kuti JO, Tipton AR (1995). Effects of nurse-plant canopy temperatures on shrub seed germination and seedling growth.. Acta Oecologica-International Journal of Ecology.

[27] Drew MB, Kirkman LK, Gholson AKJ (1998). The vascular flora of Ichauway, Baker County, Georgia: a remnant lonleaf pine/wiregrass ecosystem.. Castanea.

[28] McNab WH, Avers PE (1994). Ecological sub-regions of the United States: section descriptions Washington, DC.: USDA Forest Service Administration.. Administrative publication WO-WSA-5 Administrative publication WO-WSA-5.

[29] Goebel PC, Palik BJ, Kirkman LK, West L (1997). Field guide: landscape ecosystem types of Ichauway. Joseph W. Jones Ecological Research Center at Ichauway, Newton, Ga.. Report number 97–1 Report number 97–1.

[30] Topp GC, Davis JL, Annan AP (1980). Electromagnetic determination of soil water content: Measurements in Coaxial Transmission Lines.. Water Resources Research.

[31] Iacona G (2008). Seedling recruitment as a driver of species richness in the understory of the longleaf pine savanna [M.S. Thesis]. Gainesville: University of Florida.. 78 p.

[32] Butterfield BJ (2009). Effects of facilitation on community stability and dynamics: synthesis and future directions.. Journal of Ecology.

[33] Tielborger K, Kadmon R (2000). Temporal Environmental Variation Tips the Balance between Facilitation and Interference in Desert Plants.. Ecology.

[34] Weedon JT, Facelli JM (2008). Desert shrubs have negative or neutral effects on annuals at two levels of water availability in arid lands of South Australia.. Journal of Ecology.

[35] Manson RH, Stiles EW (1998). Links between microhabitat preferences and seed predation by small mammals in old fields.. Oikos.

[36] Cavieres LA, Badano EI (2009). Do facilitative interactions increase species richness at the entire community level?. Journal of Ecology.

